# Aerobic Versus Resistance Training Effects on Ventricular-Arterial Coupling and Vascular Function in the STRRIDE-AT/RT Trial

**DOI:** 10.3389/fcvm.2021.638929

**Published:** 2021-04-01

**Authors:** Carolyn L. Lekavich, Jason D. Allen, Daniel R. Bensimhon, Lori A. Bateman, Cris A. Slentz, Gregory P. Samsa, Aarti A. Kenjale, Brian D. Duscha, Pamela S. Douglas, William E. Kraus

**Affiliations:** ^1^Division of Cardiology, Duke University School of Medicine, Durham, NC, United States; ^2^Division of Cardiovascular Medicine, Department of Kinesiology, University of Virginia, Charlottesville, VA, United States; ^3^Cone Health and Vascular Center, Greensboro, NC, United States; ^4^Department of Biostatistics, University of North Carolina at Chapel Hill, Chapel Hill, NC, United States; ^5^Duke Clinical Research Institute, Durham, NC, United States; ^6^Duke Molecular Physiology Institute, Durham, NC, United States

**Keywords:** heart failure with preserved ejection fraction, ventricular-arterial coupling, brachial artery flow mediated dilatation, echocardiographic imaging, aerobic vs. resistance exercise training, ventricular-vascular coupling

## Abstract

**Background:** The goal was studying the differential effects of aerobic training (AT) vs. resistance training (RT) on cardiac and peripheral arterial capacity on cardiopulmonary (CP) and peripheral vascular (PV) function in sedentary and obese adults.

**Methods:** In a prospective randomized controlled trial, we studied the effects of 6 months of AT vs. RT in 21 subjects. Testing included cardiac and vascular ultrasoundography and serial CP for ventricular-arterial coupling (Ees/Ea), strain-based variables, brachial artery flow-mediated dilation (BAFMD), and peak VO_2_ (pVO_2;_ mL/kg/min) and peak O_2_-pulse (O_2_p; mL/beat).

**Results:** Within the AT group (*n* = 11), there were significant increases in rVO_2_ of 4.2 mL/kg/min (SD 0.93) (*p* = 0.001); O_2_p of 1.9 mL/beat (SD 1.3) (*p* = 0.008) and the brachial artery post-hyperemia peak diameter 0.18 mm (SD 0.08) (*p* = 0.05). Within the RT group (*n* = 10) there was a significant increase in left ventricular end diastolic volume 7.0 mL (SD 9.8; *p* = 0.05) and percent flow-mediated dilation (1.8%) (SD 0.47) (*p* = 0.004). Comparing the AT and RT groups, post exercise, rVO_2_ 2.97, (SD 1.22), (*p* = 0.03), O_2_p 0.01 (SD 1.3), (*p* = 0.01), peak hyperemic blood flow volume (1.77 mL) (SD 140.69) (*p* = 0.009), were higher in AT, but LVEDP 115 mL (SD 7.0) (*p* = 0.05) and Ees/Ea 0.68 mmHg/ml (SD 0.60) *p* = 0.03 were higher in RT.

**Discussion:** The differential effects of AT and RT in this hypothesis generating study have important implications for exercise modality and clinical endpoints.

## Highlights

- The peripheral vascular and ventricular-arterial coupling interaction needs further exploration when considering targeted AT and RT exercise interventions.
- This prospective randomized control trial identified differential effects on structural adaptations relative to exercise type which may have significant implications when considering exercise prescriptions.

## Introduction

Regular physical activity (PA) reduces the risk of cardiovascular disease, as well as cardiovascular and all-cause mortality (1–3). However, the central cardiac and peripheral vascular mechanisms whereby these significant health benefits are achieved, and how they differ with different modes of exercise remain unclear.

The effects of exercise training on left ventricular (LV) systolic function has been studied; but, the data are conflicting. Some studies show detectable improvements in LV function while others have failed to demonstrate changes (4–9). In addition, the exercise-induced effects on ventricular-arterial coupling and vascular physiology in exercise intervention clinical trials has been understudied, especially in training studies as long as 6 months (2, 9–13). Both aerobic and resistance exercise improve cardiac function; decrease arterial intima-medial thickness; and improve endothelial function, all of which are independently associated with risk of cardiovascular events and death (14–18). However, the relative effects of chronic aerobic exercise training (AT) and resistance exercise training (RT) on cardiac structural and functional, ventricular-arterial, and peripheral vascular adaptations to moderate term exercise training are not well-characterized.

As such, the purpose of this hypothesis generating sub-study of the Studies of a Targeted Risk Reduction Intervention through Defined Exercise (STRRIDE) Aerobic Training/Resistance Training (AT/RT study) (19, 20) was to compare the effects of long-term, 6 months of AT or RT (4 month control period, 2 month ramp up, 6 month training on changes in measures of cardiac and peripheral vascular morphology and function in those at elevated risk: sedentary, overweight and obese adults.

## Methods

### Study Design

More details of the study design discussed below can be obtained in published form (21).

### Subject Population

Subjects in the overall study were overweight, dyslipidemic, and sedentary men and women (exercising <1 time per week). Inclusion criteria were: age 18–70 years, body mass index 25–35 kg/m^2^, LDL-C 130–190 mg/dL or HDL-C <45 mg/dL for women <40 mg/dL for men, fasting glucose <126 mg/dL (on no medications), resting BP <160/90 mm/Hg (on no medications), and sedentary (exercise <once/week). Exclusion criteria were overt presence of cardiovascular disease, current or planned use of weight loss diet regimens, use of potentially confounding medications (e.g., hypoglycemics, anti-hypertensives), pregnancy, other metabolic or musculoskeletal diseases, unwillingness or contra-indication to undergo exercise training or study testing.

### Study Arms/Exercise Interventions

After completion of a 4-month control run-in period, subjects were randomized to one of two exercise training arms. All subjects provided verbal and written informed consent approved by the Duke University Institutional Review Board prior to participation.

Subjects were randomly assigned to either AT or RT groups using a block design for gender and race as described (22). For the AT group, subjects expended 2,000 kcal/week at 65–80% peak VO_2_ using at least two of three modalities: stationary cycling, treadmill walking or stair climbing/elliptical training. For the RT group, subjects trained using a full-body RT regimen consisting of three sessions/week of three sets of 12–15 repetitions of nine resistance exercises at 70–85% 1RM. Both AT and RT required 2 months of ramp-up, followed by 6 months of training. Repeat CPET and vascular testing was performed within 48 h following the 6 months of training. All the participants were required not to participate in any exercise outside of that prescribed in the study.

For the AT group, at each session participants wore a Polar heart rate monitor with downloadable data, to verify that the intensity and duration fell within the guidelines of the program. Aerobic compliance was calculated as percentage of completed sessions over the number of prescribed sessions per week.

For the RT group, training was prescribed as three sessions per week of three sets of 8–12 repetitions on eight Cybex machines. This consisted of four upper body and four lower body exercises, designed to target major muscle groups. Throughout the training intervention, the amount of weight lifted on each machine was increased by five pounds when the participant performed 12 repetitions with proper form on all three sets during two consecutive workout sessions. All RT sessions were verified by use of the FitLinxx Strength Training PartnerTM, a touch screen “training-partner” computer system designed to monitor and track workouts electronically. RT compliance was calculated as percentage of completed sessions over the number of prescribed sessions per week.

All post-exercise training assessments for this study—CPET testing, vascular and echocardiographic measures—were performed within 24 h after a bout of exercise and before conclusion of the 6-month training program.

*Cardiopulmonary Exercise Testing (CPET)* with a 12-lead electrocardiogram and expired gas analysis was performed on a treadmill using a TrueMax 2400 Metabolic Card. The CPET protocol consisted of 2-min stages, increasing the workload by approximately one metabolic equivalent per stage. The two greatest, consecutive, 15-s readings from each test were averaged to determine absolute VO_2_peak (L/min).

### Standard Echocardiography

Resting transthoracic echocardiograms (TTE) were obtained at two time points: (1) after a 4-month control period but prior to the initiation of a 2 month ramp-up that preceded the 6-month exercise training program and (2) at the completion of the exercise training period. Echocardiographic studies were performed on a commercially available machine with a 2.5 MHz probe and digital storage capacity (GE Vivid 7). A complete study was performed in 2-D and tissue Doppler (TDI) modes. All TDI images were obtained at a frame rate of at least 100 frames/s. 2-D measurements included LV end diastolic and end systolic volumes with calculation of LV ejection fraction (biplane Simpson's rule). Pulsed Doppler imaging was used to interrogate trans-tricuspid, trans-mitral, pulmonary venous and LV outflow tract flows. The mitral inflow E and A wave velocities, pulmonary vein systolic (S) and diastolic (D) velocities and the mitral E wave deceleration time were measured. From these, the E/A and S/D ratios were calculated. Isovolumic relaxation time (IVRT) was also determined from the mitral inflow pattern. Tei index, a measure of the combined diastolic and systolic function was also calculated with the formula (ICT + IRT)/ET where ICT represents isovolumic contraction time, IRT represents isovolumic relaxation time, and ET represents ejection time (23). For all 2D and Doppler variables, three beats were measured and the average reported. For mitral annular velocities (mitral annular peak early (Ea), late (Aa), and systolic velocities (Sa), the sample volume (6 × 6 pixels) was placed at the septal and lateral mitral annulus.

### Ventricular-Arterial Coupling

Arterial elastance (Ea), the measure of arterial elastance, was calculated as the ratio of end-systolic pressure to stroke volume (Ea = ESP/SV) (24). ESP was estimated as 0.90 multiplied times systolic blood pressure (SBP) by manual cuff at the time of echocardiogram as recommended (24). Stroke volume (SV, cm3) was measured from the left ventricular outflow tract (LVOT) diameter at the pulse wave (PW) from the echo Doppler signal. Ees (ventricular elastance) was calculated using the single-beat method outlined by Chen et al. (24).

Ees(sb) = [DBP–(Endest^*^ESP)]/(SV^*^Endest)

Endest = 0.0275 – (0.165^*^EF) + 0.3656^*^(DBP/ESP) + (0.515^*^Endavg)

Endavg = 0.35695 + (−7.2266^*^tn) + (74.249^*^tn^2^) + (−307.39^*^tn3) + (684.54^*^tn^4^) + (−856.92^*^tn^5^) + (571.95^*^tn^6^) + (−159.1^*^tn^7^)

tn (ms) = R wave to flow onset time/R wave to flow end time; determined non-invasively from the echo Doppler signal in the LV outflow tract by pulse wave (24).

### Tissue Doppler Strain Imaging Measurements

**Figure 1:**
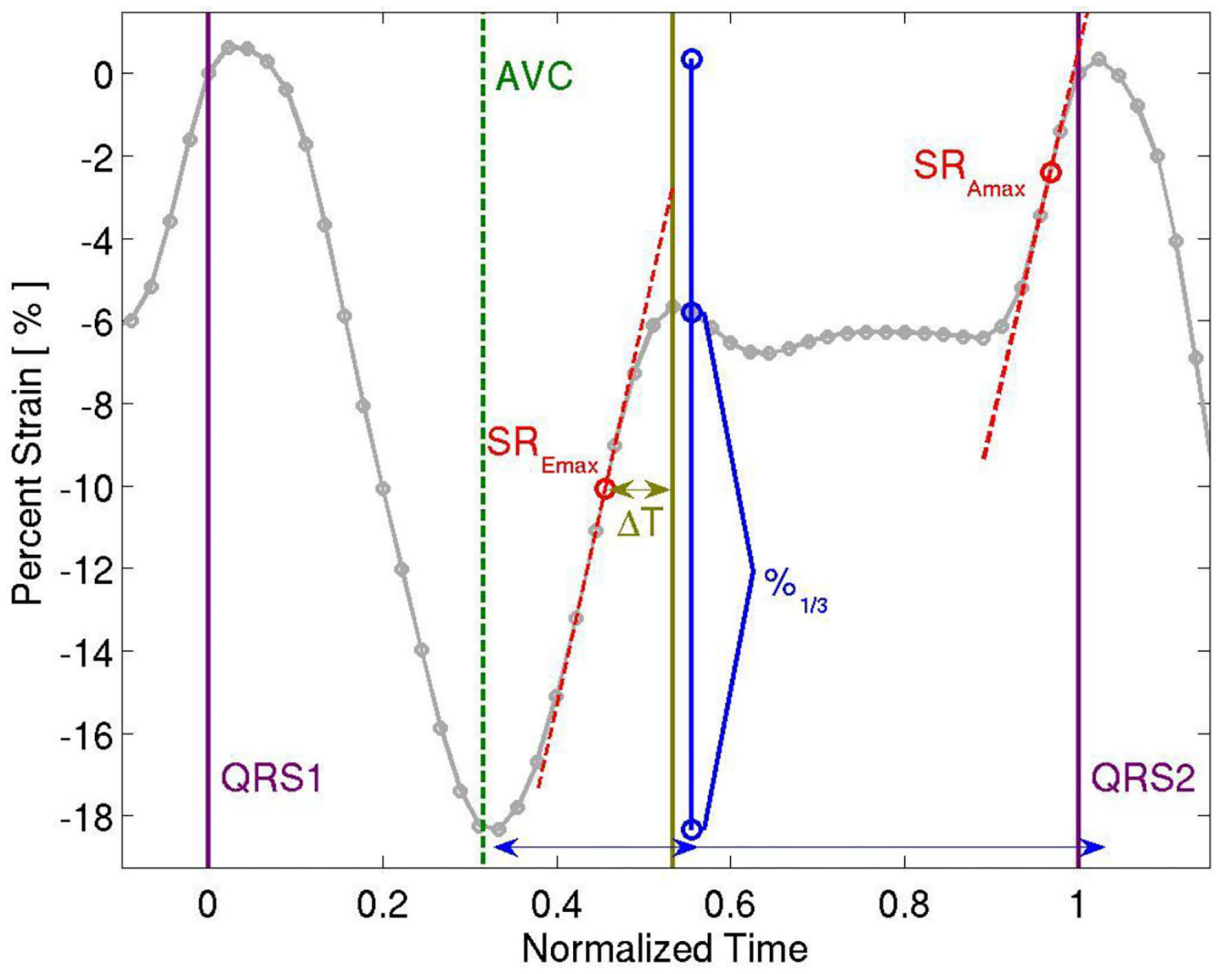
Representative Strain Curve showing time to peak longitudinal strain, peak longitudinal strain, peak early and late diastolic strain rate (SR_Emax_ and SR_Amax_, respectively), and peak diastolic strain rate deceleration time (ΔT).

Analysis of myocardial wall velocities, strain and strain rate in the apical 4-chamber view was performed offline using customized computer software (EchoPac, Version 6.3, GE). Two-dimensional speckle tracking methods were used to extract left ventricular global longitudinal strain throughout the cardiac cycle, which was analyzed for measures of systolic and diastolic function. Peak global longitudinal, circumferential and radial strains and time to peak longitudinal strain were calculated. For an evaluation of diastology, we calculated peak early and late diastolic strain rate (SR_Emax_ and SR_Amax_, respectively). In addition, peak diastolic strain rate deceleration time was calculated. See Figure 1. The echocardiograms and their analysis were performed before the current guidelines were in place and so provided somewhat limited stain information. Nevertheless, the strain derived from the 4ch view only approximates the global strain derived from the three views, especially in this instance in which each participant's compared to his or her own baseline data, acquired in the same way.

### Brachial Artery Flow Mediated Dilation

Vascular testing occurred between 7 and 10 a.m. following an overnight fast and withholding all vasoactive medications. Additionally, no exercise was performed and no alcohol or tobacco products were consumed the day before or on the morning of testing. The BAFMD technique used a high-resolution ultrasound and an 8 MHz linear array transducer (Acuson Sequoia 512) to obtain images of the artery at baseline, during and following 5 min of forearm occlusion. Ultrasound brachial artery images were obtained in longitudinal view, just proximal to the olecranon process of the elbow. With the image depth set at 3 cm, zoom and gain setting were adjusted to provide an optimal view of the center of the vessel and the anterior and posterior walls of the artery. Once the settings were optimized, they were kept constant throughout the test and the probe was stabilized with a mechanical arm. In addition, to assure maximum laminar flow, Doppler flow velocity measurements were obtained by means of range gating focused on the center of the vessel using an angle of incidence of 60 degrees. All measurements were performed using the left arm with the subject in the supine position and the forearm extended and slightly supinated. All images were recorded on a magneto-optical disk for subsequent analysis.

The forearm occlusion condition consisted of inflation of a blood pressure cuff to 50 mmHg above systolic blood pressure for a period of 5 min. The cuff was positioned ~2–3 centimeter (cm) distal to the olecranon process. Images of the brachial artery were obtained 1 min prior to inflation (baseline), during the 3rd min of inflation, and continuously for 2–3 min after cuff deflation. Throughout the brachial imaging procedure, heart rate and blood pressure were measured in order to account for any variation in central cardiovascular responses to the testing protocol. Arterial diameter and blood flow were measured from the digital recordings. Arterial diameters were determined from the anterior to posterior interface between the medial and adventitia (the “m” line), rather than between intimal layers. In addition, all diameters were end diastolic, as defined by the onset of the r-wave over at least three to four consecutive cardiac cycles using specialized imaging software (Medical Imaging Applications). The reproducibility of this technique for our group has been reported previously (25).

The nitroglycerin (NTG)-stimulated brachial artery reactivity response was tested 10 min after performing the standard BAFMD testing. Participants were given a dose of 0.4 mg of NTG sublingually and images were obtained at 5 min post-NTG administration. Participants were monitored for up to 20 min before leaving; blood pressure was continuously monitored until BP returned to pre NTG values.

Doppler forearm blood flow velocities (peak and mean) were recorded during each experiment. Brachial artery blood flow (mL/min) was estimated by multiplying the velocity-time integral of the Doppler flow signal (corrected for angle) by heart rate and the vessel cross-sectional area. The percent change in brachial artery diameter, Doppler flow velocities, and blood flow was calculated by dividing the difference from baseline values by the baseline value prior to each condition.

### Statistical Analysis

Baseline characteristics were described using mean and median for continuous variables and percentages for categorical variables. For between AT and RT groups comparisons, unpaired *T*-tests were used. For within group pre and post exercise training comparisons, paired *T*-tests were used. The across time mean vascular and physical performance measures for both AT and RT groups were analyzed using a *t*-test. Changes in strain derived variables were compared to conventional Doppler measures including the ratio of peak early (E) and late (A) diastolic mitral inflow velocities; early inflow to annular velocity ratio (E/e'); and isovolumic relaxation time (IVRT) using paired *T*-tests. To identify the association between baseline echocardiographic variables and exercise capacity, we performed univariable linear regression analyses with echocardiographic parameters as predictive variables and peak VO_2_ (pVO_2_) and peak O_2−_pulse (O_2_p) defined as peak absolute VO_2_/peak heart rate as the outcome variables. A *p*-value of 0.05 was considered significant and all statistical tests were two-sided. Statistics were done using SPSS Statistics version 22, SAS version 9.4 or JMP version 13.0. This study was conducted under the approval of the Duke Institutional Review Board.

## Results

### Baseline Characteristics

#### Demographics

Twenty-one subjects were enrolled and completed the STRRIDE-AT/RT echocardiography/peripheral vascular sub-study. For the entire cohort, the mean age was 50.0 years; 48% were men and 86% were white, the percent of participants that were male did not differ by group *X*^2^ (1, 7) = 0.12, *p* > 0.05, and the percentage of participants that are White did not differ by group *X*^2^(1, 9) = 0.59, *p* > 0.05, mean body mass index (BMI) was 30.4 kg/m^2^ and mean waist circumference was 104.3 cm. Mean systolic and diastolic blood pressures were 122.1 (SD 13.8) and 79.8 (SD 10.8) mmHg, respectively. There were no statistically significant differences between AT and RT group demographics (Table 1, AT/RT Demographics).

**Table 1:** Aerobic Training (AT) and Resistance Training (RT) demographics.

	**AT (Baseline)**	**RT (Baseline)**		**AT (Post-training)**	**RT (Post-training)**	
	**(*n* = 11)**	**(*n* = 10)**	***p*-value**	**(*n* = 11)**	**(*n* = 10)**	***p*-value**
Age (years)	53.1 (6.4)	47.0 (12.3)	0.07			
Race (% white)	81.8	90.0	0.59			
Sex (%male)	64.3	30.0	0.12			
Height (cm)	169.7 (6.8)	171.2 (9.0)	0.65			
Weight (kg)	90.5 (10.4)	86.5 (12.7)	0.41	88.9 (10.6)	86.8 (13.2)	0.68
Waist circumference cm	105.7 (8.6)	102.8 (10.1)	0.46	105.0 (8.7)	101.9 (9.0)	0.40
BMI	31.7 (3.4)	29.3 (2.3)	0.07	31.1 (3.5)	29.3 (2.2)	0.12
SBP, mmHg	122.6 (9.5)	115.4 (15.9)	0.23	124.1 (8.7)	127.1 (9.5)	0.53
DBP, mmHg	83.3 (10.1)	79.7 (10.9)	0.44	82.8 (9.5)	79.7 (10.9	0.52
HR, bpm	60.0 (9.0)	66.6 (13.0)	0.20	60.0 (9.0)	66.6 (13.0)	0.20

*All data presented as mean (SD), unless otherwise noted*.

### Baseline Echo and Exercise Capacity

#### Study Population Composition

Of the 21 subjects enrolled in the study, 11 were randomized to the AT group and 10 were randomized to the RT group. All AT and RT echocardiographic, pulsed Doppler imaging including trans tricuspid, trans mitral, pulmonary venous and LV outflow tract and vascular measures are presented in Table 2A (AT group) and Table 2B (RT group).

**Table 2A:** AT group pre- and post-training results and AT/RT pre/post-training group comparisons.

	**Baseline**	**Post-training**	**ΔTraining**	***p*-value for AT pre- and post-training**
**Exercise capacity**				
pVO_2_ mL/min	2,448 (484.9)	2,784 (483.3)	335 (74.1)	0.001^*^
mL/kg/min	26.85 (4.3)	31.09 (5.0)	4.2 (0.93)	0.001^*^
O_2_p mL/beat	14.40 (2.52)	16.34 (2.6)	1.9 (1.3)	0.008^*^
mL/kg/beat	0.16 (0.02)	0.18 (0.02)	0.02 (0.02)	0.0001^*^
Ve/VCO_2_ slope	30.09 (4.5)	30.55 (3.5)	0.46 (0.78)	0.56
**Vascular structure**				
BaseAve (mm)	3.47 (0.49)	3.63 (0.47)	0.16 (0.09)	0.11
MaxDia (mm)	3.69 (0.47)	3.86 (0.47)	0.18 (0.08)	**0.05**
%ChgMax (%)	6.45 (2.89)	6.53 (2.31)	0.09 (1.1)	0.94
TimeToMax (ms)	44.45 (14.26)64 (24.6)	45.63 (18.56)	1.18 (7.10)	0.87
Rel Ave Shear Rate (s^−1^)	0.20 (0.08)	0.19 (0.04)	−0.01 (0.09)	0.62
Rel Peak Shear Rate (s^−1^)	0.34 (0.12)	0.34 (0.11)	0.00 (0014)	0.95
NTG mediated (%)	26.2 (11.2)	23.7 (8.9)	−1.15 (11.0)	0.93
**Cardiac structure**				
Heart Rate, bpm	6.0 (9.0)	60.0 (9.0)	0 (0.0)	1.0
LVEDV, mL	95.6 (18.7)	98.8 (14.5)	4.2 (3.0)	0.20^*^
LVESV, mL	34.5 (7.4)	39.0 (8.8)	4.5(2.1)	0.06
**Systolic function**				
LVEF (%)	63.7 (4.5)	60.6 (5.4)	−3.1 (1.2)	0.62^*^
Ea (mmHg/mL)	1.6 (0.1)	1.8 (0.3)	0.2 (0.1)	0.42
Ees (mmHg/mL)	1.9 (1.2)	2.5 (1.2)	0.67 (1.2)	0.68
Ees/Ea (ratio)	1.1 (0.8)	1.3 (0.4)	0.2 (0.6)	0.77^*^
Tei Index	0.52 (0.07)	0.50 (0.07)	−0.02 (0.05)	0.14
Strain Indices				
Longitudinal global Peak Strain	−19.6 (0.64)	−17.3 (4.3)	−0.75 (5.0)	0.59
Time to peak longitudinal strain	0.40 (0.05)	0.38 (0.05)	−0.02 (0.04)	0.06
Strain rate (SR) (seconds)^−1^	−83.6 (17.6)	−82.9 (26.1)	0.71 (33.7)	0.94
Time to peak SR	0.22 (0.05)	0.18 (0.05)	−0.04 (0.07)	0.06
Circumferential peak global strain	−19.3 (6.1)	−19.2 (5.6)	0.21 (6.7)	0.91
Radial peak global strain	−26.8 (14.7)	−37.2 (22.4)	−9.84 (24.5)	0.17
**Diastolic function**				
E	0.62 (0.09)	0.66 (0.11)	0.04 (0.13)	0.31
A	0.60 (0.12)	0.62 (0.14)	0.02 (0.11)	0.58
e'	0.11 (0.02)	0.10 (0.03)	−0.004 (0.02)	0.39
E/A	1.1 (0.33)	1.1 (0.24)	0.03 (0.27)	0.72
E/e'	5.8 (1.4)	6.8 (1.7)	0.71 (1.6)	0.11
MV Dec T	196.5 (28.0)	207.0 (31.1)	10.5 (43.5)	0.38
IVRT	95.7 (11.6)	89.3 (8.4)	−6.4 (13.6)	0.10
Peak early diastolic longitudinal strain (max)	0.32 (0.08)	0.31 (0.09)	−0.001 (0.10)	0.97
Time to peak early longitudinal strain (seconds)(max)	0.16 (0.04)	0.16 (0.04)	0.002 (0.06)	0.92
SRE Max	92.8 (22.7)	101.1 (27.4)	8.2 (23.3)	0.21
SRA Max	77.4 (21.0)	78.7 (34.0)	1.3 (34.6)	0.90

* *Indicates training effect is significantly different between AT and RT*.

*pVO_2_, absolute peak VO_2_; pO_2_ pulse, absolute peak O_2_ pulse; LVEDV, center ventricular end diastolic volume; LVESV, center ventricular end systolic volume; E, early trans-mitral LV filling (meters/second); A, late transmitral LV filling (meters/second); e', average tissue Doppler velocity of the septal and mitral annulus (meters/second); MV Dec T, Deceleration time of mitral inflow Doppler; IVRT, isovolumic relaxation time; SRE Max, strain rate diastolic E maximum; SRA Max, strain rate diastolic A maximum*.

*All data presented as mean (SD), unless otherwise noted. The bold values have significant p-values*.

**Table 2B:** Characteristics pre- and post-training – RT group only.

	**Baseline**	**Post-training**	**ΔTraining**	***p*-value pre- and post-exercise**
**Exercise capacity**				
pVO_2_ mL/min	2,444 (480.3)	2,561(651.3)	117.5 (67.1)	0.11
mL/kg/min	28.3 (4.3)	29.6 (5.8)	1.3 (0.79)	0.14
O_2_p mL/beat	13.7 (3.4)	14.3 (3.9)	0.65 (1.2)	0.12
mL/kg/beat	0.16 (0.03)	0.17 (0.03)	0.01 (0.01)	0.10
Ve/VCO_2_ slope	31.1 (5.7)	32.7 (7.5)	1.6 (2.8)	0.11
**Vascular structure**				
BaseAve (mm)	3.28 (0.58)	3.33 (0.67)	0.06 (0.09)	0.54
MaxDia (mm)	3.48 (0.57)	3.59 (0.67)	0.11 (0.10)	0.30
%ChangeMax (%)	6.43 (2.16)	8.20 (2.30)	1.8 (0.47)	**0.004**
TimeToMax (ms)	40.80 (10.99)	38.20 (7.76)	−2.60 (3.49)	0.47
Rel Ave Shear Rate (s^−1^)	0.20 (0.09)	0.19 (0.08)	−0.01 (0.05)	0.54
Rel Peak Shear Rate (s^−1^)	0.35 (0.13)	0.31 (0.11)	−0.04 (0.12)	0.35
NTG mediated (%)	21.8 (7.6)	22.9 (9.0)	1.1 (2.2)	0.64
**Cardiac structure**				
Heart Rate, bpm	64.8 (10.1)	66.6 (13.0)	1.8 (7.3)	0.45
LVEDV, mL	108.9 (16.2)	115.9 (15.9)	7.0 (9.8)	**0.05**
LVESV, mL	38.7 (7.5)	42.2 (6.7)	3.5 (1.6)	0.06
**Systolic function**				
LVEF (%)	64.6 (3.6)	63.6 (4.4)	−1.0 (1.3)	0.47
Tei Index	0.50 (0.07)	0.52 (0.05)	0.01 (0.04)	0.43
Ea (mmHg/mL)	1.9 (0.5)	1.7 (0.3)	−0.2 (0.2)	0.26
Ees (mmHg/mL)	3.4 (1.2)	4.1 (1.2)	0.7 (0.9)	0.68
Ees/Ea (ratio)	1.7 (0.5)	2.3 (0.6)	0.6 (0.4)	0.29
Strain Indices				
Longitudinal global Peak Strain	−18.2 (2.9)	−18.8 (4.0)	−0.7 (5.4)	0.71
Time to peak longitudinal strain	0.41 (0.04)	0.40 (0.05)	−0.01 (0.06)	0.61
Strain rate (SR) (seconds) ^−1^	−88.3 (15.0)	−94.3 (16.1)	−5.9 (17.4)	0.31
Time to peak SR	0.22 (0.05)	0.21 (0.05)	−0.02 (0.09)	0.54
Circumferential peak global strain	−21.5 (5.2)	−20.0 (5.1)	1.6 (4.9)	0.33
Radial peak global strain	−40.6 (22.1)	−38.5 (20.3)	2.2 (23.1)	0.77
**Diastolic function**				
E	0.73 (0.14)	0.74 (0.13)	0.01 (0.04)	0.56
A	0.61 (0.17)	0.58 (0.18)	−0.03 (0.09)	0.39
e'	0.13 (0.04)	0.13 (0.03)	−0.002 (0.02)	0.76
E/A	1.37 (0.77)	1.40 (0.59)	0.03 (0.3)	0.79
E/e'	5.97 (1.27)	6.04 (1.15)	0.07 (0.98)	0.83
MV Dec T	186.3 (31.0)	198.4 (32.7)	12.1 (30.3)	0.24
IVRT	90.4 (16.2)	92.3 (15.6)	1.9 (11.0)	0.60
Peak early diastolic longitudinal strain (max)	0.26 (0.07)	0.26 (0.08)	−0.001 (0.08)	0.98
Time to peak early longitudinal strain (seconds)(max)	0.18 (0.03)	0.15 (0.04)	−0.03 (0.04)	0.04
SRE Max	115.6 (27.4)	118.6 (26.9)	3.0 (31.5)	0.77
SRA Max	68.9 (20.2)	71.6 (28.6)	2.7 (36.2)	0.82

*pVO_2_, absolute peak VO_2_; pO_2_, pulse, absolute peak O_2_ pulse; LVEDV, center ventricular end diastolic volume; LVESV, center ventricular end systolic volume; E, early trans-mitral LV filling (meters/second); A, late transmitral LV filling (meters/second); e', average tissue Doppler velocity of the septal and mitral annulus (meters/second); MV Dec T, Deceleration time of mitral inflow Doppler; IVRT, isovolumic relaxation time; SRE Max, strain rate diastolic E maximum; SRA Max, strain rate diastolic A maximum*.

*All data presented as mean (SD), unless otherwise noted. The bold values have significant p-values*.

#### Correlation of Baseline Characteristics With Exercise Capacity

Since variables of baseline cardiac structure, systolic function, and diastolic function were similar in both groups, the data were combined and evaluated across both groups for correlation with baseline exercise capacity. No measures of baseline cardiac structure or diastolic function were significantly correlated with baseline exercise capacity measured as absolute (VO_2_peak (L/min).

### Exercise Effects, Changes in Cardiac and Peripheral Vascular Structure/Function

#### Within the AT Group

A significant exercise training effect was noted, with an increase in aVO_2_ 335 mL/min (SD 74.1) (*p* = 0.001), relative VO_2_ (rVO_2)_ of 4.2 mL/kg/min (SD 0.93) (*p* = 0.001), and O_2_p 1.9 mL/beat (SD 1.3) (*p* = 0.008). Of the peripheral vascular measures, the brachial artery post-hyperemia peak diameter increased 0.18 mm, (SD 0.08), (*p* = 0.05), but due to increased resting caliber of the vessel (0.13 mm, ns) there were no changes in flow-mediated dilation 0.09%, (SD) 1.0) (*p* = 0 94). These structural changes showed a trend for post-training increased calculated peak blood flow volumes during reactive hyperemia by 104.5 ml/min (SD 168.3), (*p* = 0.06) (see Table 2A).

There was a consistent, significant correlation of time-to-peak longitudinal strain with peak VO_2_ in the AT group both pre and post training (*r* = −0.610, *p* = 0.020; *r* = −0.660, *p* = 0.010; respectively). Other measures of ventricular-arterial coupling, myocardial and chamber systolic function such as circumferential, and radial strains did not show significant changes with exercise training. There were no significant changes in traditional or strain measures of diastolic function with exercise training (see Table 2A). AT Group Pre and Post Training Results and AT/RT pre/post training group comparisons.

#### Within the RT Group

No significant exercise training effects were observed in the RT group on pVO_2_ or O_2_p but there was a significant increase in LV end diastolic volume (LVEDP) 7.0 mL (SD 9.8) (*p* = 0.05) and modest but not significant increase in LV end systolic volume (LVESV) 3.5 mL (SD 5.2) (*p* = 0.06). There were no significant changes in brachial artery resting average diameter (−0.2 mm, *p* = 0.51), however, percentage flow-mediated dilation increased 1.8 mm (SD 0.47) (*p* = 0.004). Calculated resting and peak hyperemic blood flow volumes and resting and peak brachial artery shear rates were not different from baseline following exercise training. Other measures of ventricular-arterial coupling, myocardial and chamber systolic function did not show significant changes with exercise (See Table 2B).

#### Differential Responses in the AT and RT Groups

Measures of post-training cardiac structure, systolic function, and diastolic function, peripheral vascular and ventricular-arterial coupling were analyzed for correlation with post-training exercise capacity. When comparing the post exercise mean differences between AT and RT, the mean change in the AT group was significantly higher in rVO_2_ 2.97, (SD 1.22), (*p* = 0.03), O_2_p 0.01 (SD 1.3), (*p* = 0.01), Ees/Ea 0.68 mmHg/ml (SD 0.60) *p* = 0.03 and the post-training LVEDP was higher in the RT group 7 mL (SD 3.1) (*p* = 0.05). There were no between group differences for brachial artery diameters in response to flow. However, peak hyperemic blood flow volume for the AT group was significantly greater than that of the RT group (177.8 mL) (SD 140.69) (*p* = 0.009) without any differences in shear rate. Nether group showed a significant brachial reactivity response to NTG (AT 26.2%, RT 21.8%, *p* = 0.48).

## Discussion

The unique aspects of this study illustrate significant central (cardiac) and peripheral vascular adaptations to AT and RT after 6 months of training in sedentary middle aged adults. However, the pattern of change is different. By intensively studying the coupling effects of central and peripheral vascular adaptations to moderate-length exercise training in at-risk humans, for the first time, relations among cardiorespiratory, cardiac and peripheral vascular measures have been integrated within the same study. Important new observations from this study are: (a) resistance and endurance exercise training have different but potentially congruent adaptations at the central (cardiac) and peripheral vascular levels resulting in differences in cardiorespiratory capacity, (b) with RT there was no increase in structural artery caliber or peak hyperemic blood flow or artery shear rates but there was an increase in flow mediated dilation coupled with increases in the ratio of ventricular-to-arterial elastance with no concomitant increase in cardiorespiratory capacity, (c) with AT there was an increase in peak peripheral arterial structural caliber and blood flow with no increases in shear rate of the flow-mediated dilation. This increase in arterial caliber was matched with an increase in ventricular-arterial coupling and significant increases in cardiorespiratory capacity.

This study specifically links type of exercise intervention to cardiac and vascular responses that may contribute to improved cardiopulmonary performance in middle-aged, sedentary subjects and in fact, may be synergistic, as documented by Santoro et al. (26). As expected, the study showed improvements in peak aVO_2_, peak rVO_2_, and O_2_p with AT, but not RT. Unique to this study, we observed that AT induced significant brachial artery absolute diameter and blood flow during hyperemia whereas, RT induced changes in vascular reactivity. These findings suggest harmonious adaptations to different exercise modes: AT changes vessel architecture and capacitance, whereas RT induces a stress response to exercise (vasodilation), without changing the vessel architecture (diameter).

Previous cross-sectional AT studies comparing both male (27) and female athletes (28) with healthy age-matched sedentary counterparts, report similar increases in artery caliber (29–31). Both animal and human studies, which tend to be aerobic-type trying, also demonstrate a transient increase in endothelial-mediated vasoreactivity followed by arteriogenesis in response to aerobic conditioning (25, 32, 33). However, these studies assessed changes in response to a relatively short-term training stimuli. To our knowledge, this is the first study of a longitudinal training design to observe changes in arterial structure in humans with intensive AT lasting 6 months and importantly this study included randomization to AT or RT after completion of a 4-month control run-in period.

If we consider the contrasting demands and mechanisms of blood flow delivery among AT vs. RT, this differential response is logical. Typically, RT subjects spent ~1–2 min on each set of a specific lift with a rest period between sets. This created a short period of increased blood flow and shear stress on the arterial wall. In contrast, during AT, subjects performed less intense muscular contractions for up to 40 min per session; creating an increased blood flow demand by skeletal muscle for the entire exercise bout.

When linking the vascular and cardiac measures to exercise type, we found that the LVEDP increased significantly with RT and significantly higher post-training compared to AT and the post-training mean difference in Ees/Ea was higher in RT. Given that Ees/Ea measures LV volume, force and loading effects during contraction, it reflects the ventricular-arterial interaction (24). With AT, where the resting caliber of the vessel increased and architecture changed, Ees/Ea remained balanced reflecting a central and peripheral vascular physiologic balance. This finding suggests that RT affects the ventricular-vascular balance.

In addition, unique to this study, with AT, there was a significant negative correlation between peak rVO_2_ and time-to-peak longitudinal strain (*p* = 0.001), a finding not previously reported. A shorter time to peak strain represents a more forceful myocardial contraction. There were no similar adaptations observed in the RT group. This may indicate that AT contributes to the balance of ventricular-arterial coupling while also having a training effect on myocardial contractility. Given that we observed substantive differences in the effects of AT and RT on cardiovascular structure and function, this may have important clinical implications for the use of exercise training in individuals with impaired cardiac relaxation finding, such as when considering an exercise prescription for patients with heart failure with preserved ejection fraction (HFpEF). Importantly, as physical activity interventions are tested, future studies should consider the myocardial and peripheral vascular interaction in human performance and cardiovascular health in middle-aged and elderly populations and the effects such changes may have on long-term cardiovascular health.

### Strengths and Limitations

This study has unique strengths. To the best of our knowledge, this is the first study to compare two different modes of exercise of a 6-month training program in assessments of cardiac morphology, vascular function and cardiorespiratory fitness in adults of any age or cardiovascular risk. This provides the unique opportunity to study the coupling of these components of cardiorespiratory health to two very different exercise exposures.

However, there exist also some limitations. While our older sedentary population was unique and the sample size was adequate to detect changes in cardiorespiratory physiology and fitness, in the future a larger sample size may be necessary to detect more subtle changes in vascular-arterial coupling and a more diverse study sample may provide unique phenotypic data. Our findings are thus hypothesis generating rather than definitive. In addition, all echocardiogram measurements were taken at rest as opposed to during an active state; therefore, we may have missed important dynamic myocardial adaptations that occur during submaximal and maximal exercise capacity that may include changes in ventricular-arterial coupling. Further, it is possible that, by assessing vascular changes only before and after the 6-month training program, we may have missed a critical period of physiologic and morphologic adaptation in the vascular reactivity change with AT, but captured the longer-term structural adaptations. That is, there may be a vascular reactivity change with AT, similar to that observed with 6 months of RT. This will require further investigation with measures taken at more intermediate time points.

### Implications for Clinical Practice

This study addresses differences in physiologic adaptations to endurance (AT) and resistance exercise (RT). With AT, there were significant adaptive training effects in aerobic exercise capacity, and in peripheral arterial remodeling. Furthermore, the interaction of ventricular-arterial coupling remained balanced (adaptive) with AT; but demonstrated a significant increase post exercise for the RT group. These differential findings have important implications for the use of exercise training of various modalities to achieve specific clinical endpoints.

## Data Availability Statement

The raw data supporting the conclusions of this article will be made available by the authors, without undue reservation.

## Ethics Statement

The studies involving human participants were reviewed and approved by Duke Institutional Review Board. The patients/participants provided their written informed consent to participate in this study.

## Author Contributions

WK, PD, JA, DB, and CL created the scientific conception and design of the paper. In addition, JA and AK provided key expertise to peripheral arterial physiology and measurement, PD to echocardiography, WK and BD to exercise physiology and measurement, and LB, GS, and CS to the biostatical analysis of the paper. All authors drafted, revised, authorized and made critically important contributions to the paper.

## Conflict of Interest

The authors declare that the research was conducted in the absence of any commercial or financial relationships that could be construed as a potential conflict of interest.

## Abbreviations

Ea: Arterial elastance (mmHg/ml)
BAFMD: Brachial artery flow-mediated dilation (mm)
CPET: Cardiopulmonary Exercise Testing
HFpEF: Heart failure with preserved ejection fraction
LVEDV: Left ventricular end diastolic volume
O_2_p: Oxygen pulse
pVO_2_: Peak oxygen uptake (L/min)
STRRIDE: Studies of a Targeted Risk Reduction Intervention through Defined Exercise
Ees/Ea: Ventricular-arterial coupling
Ees: Ventricular elastance (mmHg/ml).
